# The Contemporary Diagnostic Approaches to Esophageal Symptomatology

**DOI:** 10.7759/cureus.78804

**Published:** 2025-02-10

**Authors:** Amir Farah, Edoardo V Savarino, Wisam Abboud, Anna Tatakis, Amir Mari

**Affiliations:** 1 Surgery, Medical College of Wisconsin, Milwaukee, USA; 2 Surgery, Oncology, and Gastroenterology, University of Padua, Padua, ITA; 3 General Surgery, Nazareth Hospital Edinburgh Medical Missionary Society (EMMS), Nazareth, ISR; 4 General Surgery, Medical College of Wisconsin, Milwaukee, USA; 5 Gastroenterology and Hepatology, Nazareth Hospital Edinburgh Medical Missionary Society (EMMS), Nazareth, ISR

**Keywords:** achalasia, barium, diagnosis, endoscopy, esophageal symptoms, gerd, manometry, motility, reflux, symptoms

## Abstract

Esophageal symptoms, including dysphagia, heartburn, and non-cardiac chest pain, are prevalent concerns in gastroenterology. This review examines the roles of advanced diagnostic modalities such as high-resolution manometry (HRM), pH-impedance monitoring, and EndoFLIP in understanding esophageal physiology and pathology. Here, we discuss the clinical presentations of common esophageal symptoms and explore how structural abnormalities like strictures and motility disorders, including achalasia and esophageal spasms, are assessed using these tools. The diagnostic utility of endoscopy in visualizing mucosal and structural changes is highlighted alongside emerging technologies like artificial intelligence in enhancing detection capabilities. Complementary techniques, such as barium esophagrams and reflux monitoring, provide additional functional and anatomical insights, crucial for comprehensive patient evaluation. The integration of these diagnostic approaches fosters a deeper understanding of esophageal disorders, guiding effective management strategies and improving patient outcomes. This review aimed to highlight the importance of adopting a multimodal diagnostic approach in modern gastroenterological practice.

## Introduction and background

Esophageal symptoms, including dysphagia, heartburn, regurgitation, and non-cardiac chest pain, are among the most common reasons for gastroenterology consultations. These symptoms arise from a variety of structural, functional, and motility disorders, requiring accurate diagnostic approaches to guide effective management. Traditional tools like barium esophagrams and endoscopy have been complemented by advanced modalities, such as high-resolution manometry (HRM), pH-impedance monitoring, and endoluminal functional lumen imaging probe (EndoFLIP), offering unparalleled understandings of esophageal physiology. Complementary techniques, such as barium esophagrams and reflux monitoring, provide additional functional and anatomical insights, crucial for comprehensive patient evaluation. The integration of these diagnostic approaches provides a deeper understanding of esophageal disorders, guiding effective management strategies and improving patient outcomes. This review explores the clinical presentations of esophageal symptoms and examines the roles of these diagnostic modalities, highlighting their strengths, limitations, and integration in modern practice [1,2].

## Review

Clinical presentations of esophageal symptoms

Dysphagia

Dysphagia, or difficulty swallowing, is a hallmark symptom of esophageal disorders. It can be caused by structural abnormalities or motility disorders, each requiring tailored diagnostic strategies. Structural dysphagia frequently results from strictures, Schatzki’s rings, or eosinophilic esophagitis (EoE). GERD-related strictures often present with progressive dysphagia to solids and are amenable to endoscopic dilation. Schatzki’s rings, thin mucosal constrictions at the gastroesophageal junction, cause intermittent dysphagia and are easily treated with endoscopic disruption. EoE, characterized by inflammation-induced narrowing, presents distinctive findings on endoscopy, such as rings, furrows, and white exudates, with biopsies confirming eosinophilic infiltration [3,4].

Motility disorders such as achalasia, esophagogastric junction outflow obstruction (EGJ-OO), hypercontractile esophagus, and distal esophageal spasm (DES) are significant causes of dysphagia. Achalasia presents with progressive dysphagia to both solids and liquids, often accompanied by regurgitation and weight loss [5]. HRM is indispensable for diagnosing achalasia, categorizing it into three subtypes based on esophageal pressurization patterns. Type I and II achalasia, characterized by pan-esophageal pressurization, respond well to endoscopic interventions like pneumatic dilation or peroral endoscopic myotomy (POEM), while type III achalasia responds better to POEM [6]. EGJ-OO is a disorder characterized by lack or incomplete EGJ relaxation but normal esophageal motility, requiring further confirmation by esophagogram and/or EndoFLIP, and responding to endoscopic interventions [7]. DES, on the other hand, is marked by uncoordinated contractions and reduced distal latency, findings that are readily identifiable with HRM [8].

Heartburn and Regurgitation

Heartburn and regurgitation are cardinal symptoms of erosive esophagitis but also occur in non-erosive reflux disease (NERD) and functional esophageal disorders, like functional heartburn or reflux hypersensitivity [9-11]. GERD-associated heartburn is exacerbated by lying down or after meals and improves with lifestyle changes and medical treatments including proton pump inhibitors (PPI), histamine-2 receptor antagonists, potassium-competitive acid blockers, and alginate-based compounds [12-15]. Persistent symptoms despite PPI use may indicate functional heartburn or reflux hypersensitivity, requiring further evaluation with pH-impedance monitoring [16]. The Montreal definition of GERD provides a structured framework for diagnosing reflux-related disorders based on symptomatology and quality-of-life impact, and it has been recently updated by the Lyon 2 consensus, better defining the significance of actionable GERD and the role of atypical symptoms [1,17-19].

Non-cardiac Chest Pain

Non-cardiac chest pain often mimics cardiac ischemia and requires the exclusion of cardiac causes before esophageal evaluation. GERD remains a common cause, with acid-induced pain alleviated by PPI therapy [20]. Esophageal motility disorders such as DES and jackhammer esophagus also contribute to chest pain, with HRM playing a critical role in differentiating these conditions [21,22]. For instance, jackhammer esophagus is defined by elevated distal contractile integral (DCI) values, while DES features reduced distal latency and uncoordinated contractions [23,24].

Diagnostic modalities for esophageal symptoms

Endoscopy

Endoscopy plays a crucial role in the diagnostic evaluation for esophageal disorders, offering a direct view of the esophageal mucosa, structural abnormalities, and pathological changes. Its role extends beyond diagnostics to encompass advanced imaging techniques and therapeutic interventions, making it a highly versatile tool [25,26].

Structural disorders: Endoscopy is pivotal for identifying esophageal strictures, rings, webs, and other luminal narrowing. Strictures, often resulting from GERD, are visualized as concentric narrowing, and their etiology can be confirmed via biopsy [1,27]. Schatzki’s rings, mucosal constrictions at the gastroesophageal junction, are commonly associated with intermittent dysphagia and are visualized as thin, circular bands during endoscopy [28].

Barrett’s esophagus, a premalignant condition linked to chronic GERD, is diagnosed by identifying columnar-lined epithelium during endoscopy. The Prague classification quantifies the extent of Barrett’s epithelium, with the circumferential and maximal length measurements guiding surveillance intervals and assessing the risk for esophageal adenocarcinoma [1,29]. Accurate biopsies, guided by advanced imaging like narrow-band imaging (NBI), are essential for identifying dysplastic changes in Barrett’s mucosa [30].

Inflammatory disorders: Endoscopy is essential for diagnosing EoE, an immune-mediated condition often associated with food impaction and dysphagia. Classic endoscopic findings include concentric rings (trachealization), linear furrows, white exudates, edema, and friable mucosa. Biopsies showing ≥15 eosinophils per high-power field confirm the diagnosis after having excluded other causes of esophageal eosinophilia [30,31].

Malignant conditions: Endoscopy is the diagnostic modality of choice for detecting esophageal malignancies, including squamous cell carcinoma and adenocarcinoma. It allows for direct tumor visualization, staging, and tissue sampling [29]. Dysplastic lesions are often highlighted using chromoendoscopy or NBI, which enhances contrast and delineates lesion margins. Endoscopic ultrasound (EUS) further aids in determining tumor depth and regional lymph node involvement [24].

Narrow-band imaging: NBI enhances the visualization of mucosal and vascular patterns by filtering specific wavelengths of light, highlighting areas of dysplasia or early neoplasia. This is particularly beneficial in Barrett’s esophagus, where subtle dysplastic changes may otherwise go undetected [32].

Chromoendoscopy: Using dyes such as methylene blue or Lugol’s iodine, chromoendoscopy identifies abnormal mucosal patterns indicative of dysplasia or early cancer. This technique is invaluable in surveillance programs for Barrett’s esophagus and high-risk patients with squamous cell carcinoma [33].

Confocal laser endomicroscopy: Confocal laser endomicroscopy (CLE) enables real-time, high-resolution imaging of the esophageal mucosa at a cellular level. This technique has applications in Barrett’s esophagus, allowing targeted biopsies by differentiating between inflammatory and neoplastic changes [34].

Dilation of strictures: Endoscopic dilation is the first-line therapy for benign strictures, including those caused by GERD or eosinophilic esophagitis [35]. Techniques include the use of bougie dilators or balloon dilation, which mechanically widen the narrowed lumen. Dilation may also be combined with corticosteroid injections or topical therapies to prevent recurrence in EoE [30,36].

Stenting: Metal or plastic stents can be placed to palliate malignant strictures, improve swallowing, and maintain luminal patency. Stent placement is particularly useful in advanced esophageal cancers causing significant obstruction or fistula formation [24,37].

Endoscopic resection techniques and endoscopic submucosal dissection: Endoscopic mucosal resection (EMR) is used for superficial neoplastic lesions, EMR involves resecting mucosal and submucosal layers for both diagnostic and therapeutic purposes [38]. Endoscopic submucosal dissection (ESD) enables en-bloc resection of larger or deeper lesions, offering higher curative potential in early-stage esophageal cancer [39].

Management of food impaction: Endoscopy is the preferred approach for relieving food bolus impactions, particularly in conditions like EoE or Schatzki’s ring. Gentle extraction or fragmentation techniques are used to clear the obstruction without causing mucosal damage [40].

While endoscopy is indispensable for visualizing structural and mucosal abnormalities, it has limitations in evaluating esophageal motility and functional disorders. Conditions such as achalasia, DES, and ineffective esophageal motility require complementary diagnostic modalities like HRM. Similarly, EndoFLIP provides biomechanical insights into esophageal compliance and distensibility that cannot be captured via endoscopy [41,42].

Artificial intelligence (AI) in endoscopy: The integration of AI algorithms has enhanced lesion detection, particularly in Barrett’s esophagus and early esophageal cancer. AI-assisted endoscopy reduces interobserver variability and improves diagnostic accuracy, representing a significant advancement in endoscopic practice [43].

Capsule endoscopy: Though primarily used for small bowel evaluation, capsule endoscopy is a minimally invasive option for visualizing the esophagus, particularly in patients intolerant to conventional endoscopy [44].

Endoscopy, with its diagnostic, therapeutic, and emerging technological applications, remains an unparalleled tool in the management of esophageal disorders. Advances in imaging and resection techniques continue to expand its role, underscoring the importance of integrating endoscopy with complementary diagnostic tools for a comprehensive approach.

High-Resolution Manometry

High-resolution manometry (HRM) is the gold standard for evaluating esophageal motility disorders, offering unparalleled insights into esophageal pressure dynamics. By employing a closely spaced array of pressure sensors, HRM provides a detailed topographical map of esophageal motility, enhancing the diagnosis of motility abnormalities. The Chicago Classification, now in its fourth version (version 4.0), has standardized the interpretation of HRM findings, enabling consistency in diagnosing esophageal disorders across clinical centers and research studies [16,45].

Achalasia: HRM is indispensable for diagnosing achalasia, characterized by impaired relaxation of the lower esophageal sphincter (LES) and absent peristalsis. It categorizes achalasia into three subtypes based on the Chicago Classification as follows: type I, this subtype exhibits minimal esophageal pressurization, reflecting complete aperistalsis and failure of LES relaxation. Patients with type I achalasia often present with severe dysphagia and weight loss. Therapeutic approaches such as pneumatic dilation or Heller myotomy are commonly effective [46]. Type II, characterized by pan-esophageal pressurization, this subtype has the best response to therapy. Its pressurization reflects residual esophageal function and a more coordinated contractile response to retained bolus, which improves with interventions such as POEM [6,46]. Type III, this rare subtype features spastic esophageal contractions, often causing severe chest pain and dysphagia. Treatment is challenging and may include botulinum toxin injections, tailored surgical myotomies, or pharmacologic interventions targeting spasticity [46]. The progression of achalasia and treatment response can also be monitored using adjunctive tools like EndoFLIP, which measures LES distensibility during interventions [46].

Distal esophageal spasm (DES) is characterized by uncoordinated, simultaneous esophageal contractions, leading to dysphagia and non-cardiac chest pain. HRM identifies these abnormal contractions, which are defined by reduced distal latency (<4.5 seconds) and hypercontractility in the esophageal body. This disorder is often confused with achalasia due to overlapping symptoms, but HRM enables precise differentiation by documenting peristaltic behavior and LES function [46,47].

Hypercontractile esophagus: Jackhammer esophagus is another hypercontractile disorder that HRM identifies by measuring elevated distal contractile integral (DCI) values exceeding 8,000 mmHg·s·cm. These powerful contractions are often associated with chest pain and intermittent dysphagia. Therapeutic options include calcium channel blockers, botulinum toxin injections, or surgical interventions in refractory cases [48].

Ineffective esophageal motility: Ineffective esophageal motility (IEM) is diagnosed using HRM when >50% of peristaltic waves are ineffective or fail to propagate effectively. It is commonly seen in patients with GERD and is associated with poor bolus clearance and reflux symptoms. HRM findings in IEM often necessitate further evaluation using combined impedance-pH monitoring to correlate symptoms with reflux events [49,50].

Esophagogastric junction outflow obstruction: HRM has highlighted the clinical significance of esophagogastric junction outflow obstruction (EGJOO), defined as impaired LES relaxation (elevated integrated relaxation pressure) without the classic features of achalasia. This condition is heterogeneous, with etiologies ranging from subtle mechanical obstructions to functional impairments. The clinical relevance of EGJOO is assessed by integrating HRM with adjunctive tests such as EndoFLIP or timed barium esophagram [51].

Upright vs. supine positioning: Traditional HRM studies are conducted in the supine position, but upright measurements are increasingly recognized for their clinical relevance. Upright studies better simulate physiological swallowing and may improve the detection of borderline EGJOO cases [45,51].

Multiple rapid swallow (MRS) testing: Evaluates the peristaltic reserve of the esophagus by challenging it with rapid swallows. This test is particularly useful for differentiating motility disorders like IEM and assessing response to therapy in conditions such as achalasia [52].

Integrated relaxation pressure: Integrated relaxation pressure (IRP) is a critical metric in HRM, measuring the LES's ability to relax during swallowing. Elevated IRP is diagnostic of achalasia and EGJOO, while normal IRP helps exclude these disorders and guides further evaluation [47,53].

Limitations and complementary modalities: HRM’s primary limitation lies in its inability to provide real-time visualization of esophageal distensibility or bolus transit. Complementary tools like EndoFLIP and impedance-pH monitoring fill these gaps. EndoFLIP measures esophageal compliance and LES distensibility, while impedance-pH monitoring correlates reflux episodes with symptomatic events, offering a holistic understanding of esophageal function [49,54].

High-resolution impedance manometry (HRIM) combines pressure and impedance measurements to evaluate bolus transit alongside motility. This technology enhances the diagnostic yield of HRM, particularly in conditions like IEM and DES, where bolus clearance may be impaired despite normal pressure metrics [45,49]. Emerging 3D manometry systems provide a spatially detailed view of the esophagogastric junction, enabling more precise assessments of EGJ morphology and relaxation dynamics [55]. HRM’s unparalleled ability to characterize esophageal motility patterns has transformed the diagnosis and management of esophageal disorders. By integrating advanced protocols and complementary technologies, HRM continues to expand its diagnostic utility, ensuring precise and personalized patient care.

Barium Esophagram

The barium esophagram remains vital in the diagnostic evaluation of esophageal disorders, offering a non-invasive, real-time assessment of esophageal anatomy and function. Its ability to evaluate bolus transit, detect structural abnormalities, and assess esophageal emptying dynamics makes it an indispensable complement to endoscopy and HRM.

Achalasia: The barium esophagram is highly sensitive in diagnosing achalasia, with the classic "bird's beak" appearance at the lower esophageal sphincter (LES) serving as a pathognomonic finding. Timed barium esophagrams, which measure esophageal emptying at specific time intervals (e.g., 1, 2, and 5 minutes), provide quantitative metrics of esophageal clearance. These measurements are particularly valuable for assessing disease severity and monitoring treatment response in patients undergoing interventions such as pneumatic dilation, Heller myotomy, or POEM [56,57]. In cases where HRM findings are equivocal, barium esophagrams can corroborate the diagnosis by demonstrating delayed emptying and esophageal dilation.

Non-achalasia motility disorders: In motility disorders such as DES and jackhammer esophagus, barium esophagrams provide unique visualizations of esophageal contractions. DES often produces a “corkscrew” or “rosary bead” appearance due to uncoordinated, simultaneous contractions in the esophageal body. Jackhammer esophagus, on the other hand, may show vigorous peristaltic waves without specific anatomical changes. These findings complement HRM by visually correlating pressure abnormalities with functional transit issues [1,57,58].

The esophagram is instrumental in detecting subtle structural lesions that may be overlooked during endoscopy. These include the following: proximal esophageal webs and rings, thin mucosal or submucosal obstructions, particularly in the upper esophagus [59-61]. Zenker’s diverticulum, pouch-like protrusions arising from esophageal or pharyngeal walls, causing dysphagia or regurgitation [62]. Submucosal compressions, external compression by mediastinal masses or vascular anomalies, which may not distort the mucosa significantly enough to be detected on endoscopy [63].

The barium esophagram is a valuable follow-up tool for patients treated for esophageal disorders. In achalasia, it assesses the adequacy of interventions by quantifying esophageal clearance and detecting residual narrowing or obstruction. In patients with GERD treated with fundoplication, the study can identify complications such as wrap slippage or tightness, which may cause dysphagia or obstruction [59-61,64].

Advantages: Real-time bolus transit evaluation - unlike HRM, which primarily measures pressure changes, the esophagram visually tracks bolus movement, identifying areas of delayed or impaired transit. [64]. Accessibility and speed - this test is widely available, relatively inexpensive, and can be performed quickly, making it a practical choice for initial evaluation in resource-limited settings. Detection of subtle abnormalities - barium esophagrams are particularly sensitive to conditions such as small diverticula or webs, which may be missed on endoscopy [59,64].

Limitations: Lack of pressure dynamics assessment - this test does not provide information on esophageal pressure dynamics or LES relaxation, areas where HRM excels [57]. Radiation exposure - while the radiation dose is relatively low, repeated studies should be avoided, especially in younger patients or those requiring long-term follow-up [57]. Limited sensitivity for early disease - subtle motility disorders or functional abnormalities, particularly in the early stages, may not produce detectable changes in barium studies. For such cases, HRM or EndoFLIP may be more diagnostic [51].

Recent advancements in fluoroscopic technology have enhanced the diagnostic potential of the barium esophagram. High-resolution fluoroscopy offers improved image clarity, enabling better visualization of subtle structural or functional changes [65]. Quantitative metrics - algorithms for analyzing esophageal transit times and bolus retention are under development, potentially standardizing the interpretation of barium esophagram findings [45]. The ability of the barium esophagram to complement findings from endoscopy and HRM ensures a comprehensive evaluation of both structural and functional abnormalities, guiding effective management.

Reflux Monitoring

Ambulatory pH monitoring is the gold standard for diagnosing GERD, particularly in patients with atypical or refractory symptoms. It provides objective measurements of esophageal acid exposure and evaluates the correlation between symptoms and reflux events, offering a critical analysis of the etiology of reflux-related complaints.

Quantifying acid exposure: The pH monitoring quantifies esophageal acid exposure by calculating the percentage of time the esophageal pH falls below 4. This metric, known as the acid exposure time (AET), is a cornerstone of GERD diagnosis. An AET >6% is considered abnormal, while values between 4% and 6% are borderline and require additional context from symptom-reflux association indices such as the symptom index (SI) and symptom association probability (SAP) [42,66]. The clinical usefulness of this measurement has been confirmed in patients with typical and atypical symptoms [18,67-69].

Symptom-reflux correlation: Combined pH-impedance monitoring enhances diagnostic accuracy by distinguishing between acid and non-acid reflux events, which are common contributors to persistent symptoms despite proton pump inhibitor therapy. Indices like the SI and SAP evaluate the temporal relationship between reflux episodes and symptoms, helping differentiate GERD from functional heartburn or reflux hypersensitivity syndromes [70,71]. Functional heartburn, characterized by normal acid exposure and a lack of symptom-reflux correlation, often requires alternative therapeutic strategies including neuromodulators [2,16].

Non-acid reflux: The inclusion of impedance measurements with pH monitoring allows the detection of weakly acidic or non-acid reflux episodes that would otherwise go unnoticed with standard pH monitoring. This is particularly valuable in patients with persistent symptoms despite adequate acid suppression, enabling tailored treatment approaches [72-74].

Mucosal permeability: Esophageal mucosal permeability has been correlated with the measurements of impedance baseline during impedance-reflux monitoring, allowing data collection on mucosal impairment. This has been associated with reflux disease diagnosis and response to medical or surgical therapy [68,75-77].

Pre-surgical evaluation: The pH monitoring is indispensable in patients being considered for anti-reflux surgery, such as fundoplication. Confirming abnormal acid exposure is critical to ensure appropriate patient selection and optimize surgical outcomes. Wireless pH monitoring systems, such as the Bravo capsule, are particularly useful in these settings, providing extended monitoring durations that capture day-to-day variability in reflux patterns [42,78,79].

Wireless pH monitoring systems: The Bravo capsule is a wireless pH monitoring system that adheres to the esophageal mucosa, transmitting real-time pH data to an external receiver for up to 96 hours. This extended monitoring period increases diagnostic sensitivity by accounting for variability in reflux patterns over several days. It also enhances patient comfort by eliminating the need for nasoesophageal catheters, which can interfere with normal eating and activity patterns [80,81].

Combined pH-impedance monitoring: This technology simultaneously measures pH and impedance, providing a comprehensive view of esophageal reflux patterns. Impedance measurements track bolus transit and identify weakly acidic or non-acid reflux episodes, enabling more precise symptom correlation and differentiation of GERD subtypes. For patients with refractory symptoms, combined pH-impedance monitoring is invaluable for distinguishing between persistent acid reflux, non-acid reflux, and functional disorders [50,80,81].

Automated analysis algorithms: Recent advancements in pH monitoring include automated software tools that analyze pH and impedance data, reducing interobserver variability and enhancing diagnostic accuracy. These tools generate detailed reports highlighting clinically significant events, improving the efficiency of pH monitoring interpretation [45,82].

Limitations: Inability to evaluate motility - pH monitoring focuses solely on acid exposure and reflux events, offering no information about esophageal motility or LES pressure dynamics. Conditions like achalasia, diffuse esophageal spasm, or esophagogastric junction outflow obstruction may mimic GERD but require complementary testing with HRM for accurate diagnosis [83,84].

Overlap with functional disorders: In patients with normal acid exposure but persistent symptoms, pH monitoring may not differentiate functional heartburn from reflux hypersensitivity. These conditions necessitate additional testing or empiric therapeutic trials to clarify the diagnosis [42,84].

Technical challenges: Traditional catheter-based systems can cause discomfort, limiting patient compliance and potentially altering normal eating or sleeping patterns. While wireless systems address these issues, their higher cost and limited availability may restrict widespread use [83].

Integration with other modalities: For a comprehensive evaluation of esophageal symptoms, pH monitoring is often combined with HRM and endoscopy. HRM identifies motility disorders and esophagogastric junction abnormalities that may contribute to reflux symptoms. Endoscopy visualizes mucosal damage, structural abnormalities, and Barrett’s esophagus, providing complementary insights into GERD-related complications [41].

The advent of advanced pH monitoring techniques, including wireless systems and combined impedance-pH monitoring, has significantly enhanced the diagnostic accuracy for GERD and related disorders. These technologies enable a nuanced understanding of reflux patterns and their relationship to symptoms, ensuring precise diagnosis and tailored treatment strategies.

EndoFLIP

EndoFLIP represents a significant advancement in assessing esophageal compliance and distensibility [85]. Unlike HRM, which evaluates pressure dynamics, EndoFLIP provides biomechanical insights into LES relaxation and esophageal wall stiffness.

In achalasia, EndoFLIP measures the distensibility index (DI), offering quantitative data on LES relaxation and esophageal compliance. This is particularly valuable in cases with borderline HRM findings or EGJOO. During surgical procedures like POEM or Heller myotomy, EndoFLIP guides intraoperative decisions by confirming the adequacy of LES disruption, reducing the risk of persistent dysphagia [86]. In eosinophilic esophagitis, EndoFLIP evaluates esophageal stiffness, reflecting the degree of fibrosis or inflammatory remodeling. Its role in assessing esophageal compliance in other conditions, such as functional esophageal disorders, is also emerging [87]. The limitations of EndoFLIP lie in its availability, as it is predominantly used in specialized centers and its reliance on operator expertise for accurate interpretation. Nevertheless, its ability to complement HRM and endoscopy makes it a valuable tool in the comprehensive evaluation of esophageal disorders [88].

## Conclusions

The evaluation of esophageal symptoms has been significantly enhanced by advances in diagnostic modalities. Endoscopy remains the foundation for assessing structural abnormalities, while HRM is essential for diagnosing motility disorders. Complementary tools like barium esophagrams and reflux testing provide functional and anatomical insights, while EndoFLIP offers novel biomechanical evaluations. Together, these modalities create a comprehensive diagnostic framework, allowing for the precise identification of esophageal pathologies and the development of tailored treatment approaches.

## References

[1] Vakil N, van Zanten SV, Kahrilas P, Dent J, Jones R (2006). The Montreal definition and classification of gastroesophageal reflux disease: a global evidence-based consensus. Am J Gastroenterol.

[2] Savarino E, de Bortoli N, De Cassan C (2017). The natural history of gastro-esophageal reflux disease: a comprehensive review. Dis Esophagus.

[3] de Bortoli N, Visaggi P, Penagini R (2024). The 1st EoETALY consensus on the diagnosis and management of eosinophilic esophagitis - current treatment and monitoring. Dig Liver Dis.

[4] de Bortoli N, Visaggi P, Penagini R (2024). The 1st EoETALY consensus on the diagnosis and management of eosinophilic esophagitis - definition, clinical presentation and diagnosis. Dig Liver Dis.

[5] Savarino E, Bhatia S, Roman S, Sifrim D, Tack J, Thompson SK, Gyawali CP (2022). Achalasia. Nat Rev Dis Primers.

[6] Provenza CG, Romanelli JR (2025). Achalasia: diagnosis and management. Surg Clin North Am.

[7] Bach L, Vela MF (2024). Esophagogastric junction outflow obstruction (EGJOO): a manometric phenomenon or clinically impactful problem. Curr Gastroenterol Rep.

[8] Kwiatek MA, Hirano I, Kahrilas PJ, Rothe J, Luger D, Pandolfino JE (2011). Mechanical properties of the esophagus in eosinophilic esophagitis. Gastroenterology.

[9] Marabotto E, Pasta A, Calabrese F, Ribolsi M, Mari A, Savarino V, Savarino EV (2024). The clinical spectrum of gastroesophageal reflux disease: facts and fictions. Visc Med.

[10] Savarino V, Marabotto E, Zentilin P (2020). Esophageal reflux hypersensitivity: non-GERD or still GERD?. Dig Liver Dis.

[11] Savarino E, Zentilin P, Savarino V (2013). NERD: an umbrella term including heterogeneous subpopulations. Nat Rev Gastroenterol Hepatol.

[12] Mari A, Marabotto E, Ribolsi M, Zingone F, Barberio B, Savarino V, Savarino EV (2023). Encouraging appropriate use of proton pump inhibitors: existing initiatives and proposals for the future. Expert Rev Clin Pharmacol.

[13] Barberio B, Visaggi P, Savarino E, de Bortoli N, Black CJ, Ford AC (2023). Comparison of acid-lowering drugs for endoscopy negative reflux disease: systematic review and network meta-analysis. Neurogastroenterol Motil.

[14] Hungin AP, Yadlapati R, Anastasiou F (2024). Management advice for patients with reflux-like symptoms: an evidence-based consensus. Eur J Gastroenterol Hepatol.

[15] Pasta A, Pelizzaro F, Marabotto E (2024). Patient journey in gastroesophageal reflux disease: real-world perspectives from Italian gastroenterologists, primary care physicians, and ENT specialists. Therap Adv Gastroenterol.

[16] Yadlapati R, Gyawali CP, Pandolfino JE (2022). AGA clinical practice update on the personalized approach to the evaluation and management of GERD: expert review. Clin Gastroenterol Hepatol.

[17] Gyawali CP, Yadlapati R, Fass R (2024). Updates to the modern diagnosis of GERD: Lyon consensus 2.0. Gut.

[18] Calabrese F, Pasta A, Bodini G (2024). Applying Lyon consensus criteria in the work-up of patients with extra-oesophageal symptoms - a multicentre retrospective study. Aliment Pharmacol Ther.

[19] Gyawali CP, Marchetti L, Rogers BD (2024). The Lyon score: a novel reflux scoring system based on the Lyon consensus 2.0 that associates with treatment outcome from antireflux therapy. Am J Gastroenterol.

[20] Ribolsi M, Marchetti L, Olmi LM, Cicala M, Savarino E (2025). Esophageal chest pain resembles heartburn in reflux metrics and response to proton pump inhibitor therapy. Neurogastroenterol Motil.

[21] Chen JW, Savarino E, Smout A, Xiao Y, de Bortoli N, Yadlapati R, Cock C (2021). Chicago classification update (v4.0): technical review on diagnostic criteria for hypercontractile esophagus. Neurogastroenterol Motil.

[22] de Bortoli N, Gyawali PC, Roman S (2021). Hypercontractile esophagus from pathophysiology to management: proceedings of the Pisa symposium. Am J Gastroenterol.

[23] Pandolfino JE, Richter JE, Ours T, Guardino JM, Chapman J, Kahrilas PJ (2003). Ambulatory esophageal pH monitoring using a wireless system. Am J Gastroenterol.

[24] Patel DA, Yadlapati R, Vaezi MF (2022). Esophageal motility disorders: current approach to diagnostics and therapeutics. Gastroenterology.

[25] Fornari F, Wagner R (2012). Update on endoscopic diagnosis, management and surveillance strategies of esophageal diseases. World J Gastrointest Endosc.

[26] Farah A, Tatakis A, Malshy K, Mahajna A, Sayida S (2024). Real-time perfusion and leak assessment in bariatric surgery: bridging traditional and advanced techniques. Cureus.

[27] Adkins C, Takakura W, Spiegel BM, Lu M, Vera-Llonch M, Williams J, Almario CV (2020). Prevalence and characteristics of dysphagia based on a population-based survey. Clin Gastroenterol Hepatol.

[28] Dellon ES, Liacouras CA, Molina-Infante J (2018). Updated international consensus diagnostic criteria for eosinophilic esophagitis: proceedings of the AGREE conference. Gastroenterology.

[29] Dellon ES, Hirano I (2018). Epidemiology and natural history of eosinophilic esophagitis. Gastroenterology.

[30] Gómez-Aldana A, Jaramillo-Santos M, Delgado A, Jaramillo C, Lúquez-Mindiola A (2019). Eosinophilic esophagitis: current concepts in diagnosis and treatment. World J Gastroenterol.

[31] Hirano I, Furuta GT (2020). Approaches and challenges to management of pediatric and adult patients with eosinophilic esophagitis. Gastroenterology.

[32] Barbeiro S, Libânio D, Castro R, Dinis-Ribeiro M, Pimentel-Nunes P (2018). Narrow-band imaging: clinical application in gastrointestinal endoscopy. GE Port J Gastroenterol.

[33] Davila RE (2009). Chromoendoscopy. Gastrointest Endosc Clin N Am.

[34] Pilonis ND, Januszewicz W, di Pietro M (2022). Confocal laser endomicroscopy in gastro-intestinal endoscopy: technical aspects and clinical applications. Transl Gastroenterol Hepatol.

[35] Burr NE, Everett SM (2019). Management of benign oesophageal strictures. Frontline Gastroenterol.

[36] Lew RJ, Kochman ML (2002). A review of endoscopic methods of esophageal dilation. J Clin Gastroenterol.

[37] Monte Junior ES, de Moura DT, Ribeiro IB (2021). Endoscopic vacuum therapy versus endoscopic stenting for upper gastrointestinal transmural defects: systematic review and meta-analysis. Dig Endosc.

[38] Herman T, Megna B, Pallav K, Bilal M (2023). Endoscopic mucosal resection: tips and tricks for gastrointestinal trainees. Transl Gastroenterol Hepatol.

[39] Draganov PV, Aihara H, Karasik MS (2021). Endoscopic submucosal dissection in North America: a large prospective multicenter study. Gastroenterology.

[40] Redd WD, McCallen JD, Xue Z (2024). Association between time from esophageal food impaction to endoscopy and adverse events. Gastrointest Endosc.

[41] Delshad SD, Almario CV, Chey WD, Spiegel BM (2020). Prevalence of gastroesophageal reflux disease and proton pump inhibitor-refractory symptoms. Gastroenterology.

[42] Gyawali CP, Carlson DA, Chen JW, Patel A, Wong RJ, Yadlapati RH (2020). ACG clinical guidelines: clinical use of esophageal physiologic testing. Am J Gastroenterol.

[43] Okagawa Y, Abe S, Yamada M, Oda I, Saito Y (2022). Artificial intelligence in endoscopy. Dig Dis Sci.

[44] Papaefthymiou A, Koffas A, Laskaratos FM, Epstein O (2022). Upper gastrointestinal video capsule endoscopy: the state of the art. Clin Res Hepatol Gastroenterol.

[45] Fox MR, Sweis R, Yadlapati R (2021). Chicago classification version 4.0© technical review: update on standard high-resolution manometry protocol for the assessment of esophageal motility. Neurogastroenterol Motil.

[46] Kahrilas PJ, Carlson DA, Pandolfino JE (2023). Advances in the diagnosis and management of achalasia and achalasia-like syndromes: insights from HRM and FLIP. Gastro Hep Adv.

[47] van Hoeij FB, Bredenoord AJ (2016). Clinical application of esophageal high-resolution manometry in the diagnosis of esophageal motility disorders. J Neurogastroenterol Motil.

[48] Clément M, Zhu WJ, Neshkova E, Bouin M (2019). Jackhammer esophagus: from manometric diagnosis to clinical presentation. Can J Gastroenterol Hepatol.

[49] Gyawali CP, Sifrim D, Carlson DA (2019). Ineffective esophageal motility: concepts, future directions, and conclusions from the Stanford 2018 symposium. Neurogastroenterol Motil.

[50] Kamboj AK, Katzka DA, Vela MF, Yadlapati R, Ravi K (2024). A practical approach to ineffective esophageal motility. Neurogastroenterol Motil.

[51] Triggs JR, Carlson DA, Beveridge C, Jain A, Tye MY, Kahrilas PJ, Pandolfino JE (2019). Upright integrated relaxation pressure facilitates characterization of esophagogastric junction outflow obstruction. Clin Gastroenterol Hepatol.

[52] Shaker A, Stoikes N, Drapekin J, Kushnir V, Brunt LM, Gyawali CP (2013). Multiple rapid swallow responses during esophageal high-resolution manometry reflect esophageal body peristaltic reserve. Am J Gastroenterol.

[53] Ghisa M, Laserra G, Marabotto E (2021). Achalasia and obstructive motor disorders are not uncommon in patients with eosinophilic esophagitis. Clin Gastroenterol Hepatol.

[54] Donnan EN, Pandolfino JE (2020). EndoFLIP in the esophagus: assessing sphincter function, wall stiffness, and motility to guide treatment. Gastroenterol Clin North Am.

[55] Kwiatek MA, Pandolfino JE, Kahrilas PJ (2011). 3D-high resolution manometry of the esophagogastric junction. Neurogastroenterol Motil.

[56] Vaezi MF, Felix VN, Penagini R (2016). Achalasia: from diagnosis to management. Ann N Y Acad Sci.

[57] Pandolfino JE, Gawron AJ (2015). Achalasia: a systematic review. JAMA.

[58] Vaezi MF, Pandolfino JE, Yadlapati RH, Greer KB, Kavitt RT (2020). ACG clinical guidelines: diagnosis and management of achalasia. Am J Gastroenterol.

[59] O'Rourke AK, Lazar A, Murphy B, Castell DO, Martin-Harris B (2016). Utility of esophagram versus high-resolution manometry in the detection of esophageal dysmotility. Otolaryngol Head Neck Surg.

[60] Hawkins D, Cabrera CI, Kominsky R, Nahra A, Howard NS, Maronian N (2021). Dysphagia evaluation: the added value of concurrent MBS and esophagram. Laryngoscope.

[61] Neyaz Z, Gupta M, Ghoshal UC (2013). How to perform and interpret timed barium esophagogram. J Neurogastroenterol Motil.

[62] Banerjee D, Magnelli LL, Oliva M (2022). Characterizing a CT esophagram protocol after flexible endoscopic diverticulotomy for Zenker's diverticulum: a retrospective series. Transl Gastroenterol Hepatol.

[63] Levine MS, Rubesin SE (2005). Diseases of the esophagus: diagnosis with esophagography. Radiology.

[64] Blonski W, Kumar A, Feldman J, Richter JE (2018). Timed barium swallow: diagnostic role and predictive value in untreated achalasia, esophagogastric junction outflow obstruction, and non-achalasia dysphagia. Am J Gastroenterol.

[65] Scharitzer M, Pokieser P, Ekberg O (2024). Oesophageal fluoroscopy in adults - when and why?. Br J Radiol.

[66] Sharma P, Yadlapati R (2021). Pathophysiology and treatment options for gastroesophageal reflux disease: looking beyond acid. Ann N Y Acad Sci.

[67] Frazzoni M, Frazzoni L, Ribolsi M (2022). Applying Lyon Consensus criteria in the work-up of patients with proton pump inhibitory-refractory heartburn. Aliment Pharmacol Ther.

[68] Frazzoni L, Frazzoni M, De Bortoli N (2022). Application of Lyon Consensus criteria for GORD diagnosis: evaluation of conventional and new impedance-pH parameters. Gut.

[69] Ribolsi M, Savarino E, De Bortoli N (2014). Reflux pattern and role of impedance-pH variables in predicting PPI response in patients with suspected GERD-related chronic cough. Aliment Pharmacol Ther.

[70] Frazzoni M, de Bortoli N, Frazzoni L (2017). Impairment of chemical clearance and mucosal integrity distinguishes hypersensitive esophagus from functional heartburn. J Gastroenterol.

[71] Savarino E, Zentilin P, Tutuian R, Pohl D, Gemignani L, Malesci A, Savarino V (2012). Impedance-pH reflux patterns can differentiate non-erosive reflux disease from functional heartburn patients. J Gastroenterol.

[72] Frazzoni M, Frazzoni L, Ribolsi M, Russo S, Conigliaro R, De Bortoli N, Savarino E (2023). On-therapy impedance-pH monitoring can efficiently characterize PPI-refractory GERD and support treatment escalation. Neurogastroenterol Motil.

[73] Frazzoni M, de Bortoli N, Frazzoni L (2017). The added diagnostic value of postreflux swallow-induced peristaltic wave index and nocturnal baseline impedance in refractory reflux disease studied with on-therapy impedance-pH monitoring. Neurogastroenterol Motil.

[74] Savarino E, Zentilin P, Tutuian R (2008). The role of nonacid reflux in NERD: lessons learned from impedance-pH monitoring in 150 patients off therapy. Am J Gastroenterol.

[75] Ribolsi M, Frazzoni M, Marabotto E (2021). Novel impedance-pH parameters are associated with proton pump inhibitor response in patients with inconclusive diagnosis of gastro-oesophageal reflux disease according to Lyon Consensus. Aliment Pharmacol Ther.

[76] Frazzoni M, Frazzoni L, Tolone S, De Bortoli N, Savarino V, Savarino E (2018). Lack of improvement of impaired chemical clearance characterizes PPI-refractory reflux-related heartburn. Am J Gastroenterol.

[77] Frazzoni L, Frazzoni M, de Bortoli N (2017). Postreflux swallow-induced peristaltic wave index and nocturnal baseline impedance can link PPI-responsive heartburn to reflux better than acid exposure time. Neurogastroenterol Motil.

[78] Savarino E, Frazzoni M, Marabotto E (2020). A SIGE-SINGEM-AIGO technical review on the clinical use of esophageal reflux monitoring. Dig Liver Dis.

[79] Salvador R, Pandolfino JE, Costantini M (2025). The role of high-resolution manometry before and following antireflux surgery: the Padova Consensus. Ann Surg.

[80] Zeki SS, Miah I, Visaggi P (2023). Extended wireless pH monitoring significantly increases gastroesophageal reflux disease diagnoses in patients with a normal pH impedance study. J Neurogastroenterol Motil.

[81] Hasak S, Yadlapati R, Altayar O (2020). Prolonged wireless pH monitoring in patients with persistent reflux symptoms despite proton pump inhibitor therapy. Clin Gastroenterol Hepatol.

[82] Zhou MJ, Zikos T, Goel K (2023). Development and validation of a machine learning system to identify reflux events in esophageal 24-hour pH/impedance studies. Clin Transl Gastroenterol.

[83] Korszun K, Dyrla P, Wojtuń S, Gil J (2014). The diagnostic impact and limitations of 24 hour pH monitoring with multichannel intraluminal impedance. [Article in Polish]. Pol Merkur Lekarski.

[84] Di Fiore JM, Arko M, Churbock K, Hibbs AM, Martin RJ (2009). Technical limitations in detection of gastroesophageal reflux in neonates. J Pediatr Gastroenterol Nutr.

[85] Savarino E, di Pietro M, Bredenoord AJ (2020). Use of the functional lumen imaging probe in clinical esophagology. Am J Gastroenterol.

[86] Mari A, Brun R, Shibli F, Yishai R, Dickman R (2024). Advances on esophageal measurements: high resolution manometry, timed barium swallow, endoflip and PH monitoring. [Article in Hebrew]. Harefuah.

[87] Hoffmann NV, Keeley K, Wechsler JB (2023). Esophageal distensibility defines fibrostenotic severity in pediatric eosinophilic esophagitis. Clin Gastroenterol Hepatol.

[88] Casabona-Francés S, Sanz-García A, Ortega GJ (2024). A new method to evaluate lower esophageal distension capacity in eosinophilic esophagitis by using functional lumen imaging probe (EndoFLIP™). Diagnostics (Basel).

